# Variations in the Processing of DNA Double-Strand Breaks Along 60-MeV Therapeutic Proton Beams

**DOI:** 10.1016/j.ijrobp.2015.07.2279

**Published:** 2016-05-01

**Authors:** Pankaj Chaudhary, Thomas I. Marshall, Frederick J. Currell, Andrzej Kacperek, Giuseppe Schettino, Kevin M. Prise

**Affiliations:** ∗ Centre for Cancer Research and Cell Biology, Queen's University Belfast, Belfast, United Kingdom; † Centre for Plasma Physics, School of Mathematics and Physics, Queen's University Belfast, Belfast, United Kingdom; ‡ Douglas Cyclotron, Clatterbridge Cancer Centre, Bebbington, Wirral, United Kingdom; § National Physical Laboratory, Teddington, United Kingdom

## Abstract

**Purpose:**

To investigate the variations in induction and repair of DNA damage along the proton path, after a previous report on the increasing biological effectiveness along clinically modulated 60-MeV proton beams.

**Methods and Materials:**

Human skin fibroblast (AG01522) cells were irradiated along a monoenergetic and a modulated spread-out Bragg peak (SOBP) proton beam used for treating ocular melanoma at the Douglas Cyclotron, Clatterbridge Centre for Oncology, Wirral, Liverpool, United Kingdom. The DNA damage response was studied using the 53BP1 foci formation assay. The linear energy transfer (LET) dependence was studied by irradiating the cells at depths corresponding to entrance, proximal, middle, and distal positions of SOBP and the entrance and peak position for the pristine beam.

**Results:**

A significant amount of persistent foci was observed at the distal end of the SOBP, suggesting complex residual DNA double-strand break damage induction corresponding to the highest LET values achievable by modulated proton beams. Unlike the directly irradiated, medium-sharing bystander cells did not show any significant increase in residual foci.

**Conclusions:**

The DNA damage response along the proton beam path was similar to the response of X rays, confirming the low-LET quality of the proton exposure. However, at the distal end of SOBP our data indicate an increased complexity of DNA lesions and slower repair kinetics. A lack of significant induction of 53BP1 foci in the bystander cells suggests a minor role of cell signaling for DNA damage under these conditions.

**Summary**

Residual DNA DSB damage contributes to late normal tissue toxicity. Here we studied the variations in DNA DSB damage processing along and in the surroundings of therapeutic proton beams in normal human cells using the 53BP1 foci assay. Our results indicate a significant induction of complex DNA damage at the distal end of the Bragg peak. Variation in the DNA repair efficiency is important for optimization of proton therapy combined with DNA repair inhibitors.

## Introduction

Radiation therapy relies on induction of critical levels of DNA damage in the tumor cells, leading to apoptosis, necrosis, and mitotic cell death (1). Recent technological advances make it now possible to treat tumors more precisely than before using spatially and temporally modulated beams (2). Protons, with their superior depth–dose deposition properties over photons, might offer an advantage for treatment of tumors near critical organs (3). In proton therapy, a constant RBE (relative biological effectiveness) value of 1.1 is used to design treatment plans (4). This, however, represents an average, because a rapid drop in the proton energy and steep rise of the linear energy transfer (LET) are expected toward the distal end of the Bragg curve, resulting in an experimentally observed increase in effectiveness and therefore variable RBE (5). Several investigators, including our group, have shown the potential clinical impact of the adoption of a variable RBE 6, 7. Photons induce uniform damage along the depth, and the total absorbed dose can be used to define the response, whereas charged particles induce nonuniform damage along the track: the complexity of DNA lesions increases with the slowing down of the particle owing to the clustering of ionization events (8). Linear energy transfer–dependent changes in DNA damage and subsequent repair have been well reported (9). Although most DNA damage induced by low-LET radiation can be efficiently repaired, high-LET radiations are associated with increased formation of repair-refractory clustered DNA lesions, misrepaired double-strand breaks (DSBs), and exchange-type chromosomal aberrations, leading to increased cellular lethality (10). Because the LET for protons varies along the particle path, a simple assumption of uniform DNA damage and repair similar to that experienced after X ray exposure may not be justified. Although better dose conformation and higher precision than with photon beams are the key advantages of using proton therapy, the quality of the DNA damage induced and its impact on the cell repair efficiency must also be considered to optimize the treatments (11).

Residual DSB damage (ie, unrepaired DSBs at 24 hours after irradiation), along with a tissue-dependent cascade of biochemical processes, plays an important role in late normal tissue response, and many investigators have highlighted the role of persistent DSB foci as late normal tissue toxicity biomarkers 12, 13, 14, 15. Moreover, radiation therapy is often applied in combination with pharmaceutical agents, which target the DNA repair mechanisms of cancerous cells with the aim of increasing radiation effectiveness. Complexity of DNA lesions has been shown to play a key role for selection and activation of repair pathways and tissue response mechanisms 16, 17, 18, 19. For monoenergetic proton beams, the LET values reach >35 keV/μm (20), with approximately 3% of the total dose delivered by >20 keV/μm LET component (21). Values exceeding 5 keV/μm are being considered of potential clinical interest (11). This is of particular concern because the high-LET dose component is at the end of the proton path and therefore in close proximity to healthy tissue (22). Further understanding of the quality of proton-induced DNA damage is therefore required for the optimization of the treatment plans, particularly when used in combination with DNA repair inhibitors and cancer cell–specific chemical agents (ie, poly ADP ribose polymerase and Ape-1/ref-1).

Several investigators have measured the DNA damage response at various positions in the Bragg curve using plasmid DNA and mammalian cell lines 23, 24. However, the depth resolution and positional accuracy reported is of a few millimeters, which results in an average LET investigation overlooking the spatial dynamics of the DNA damage response at the sub-millimeter scale. Data on DNA damage response within the spread-out Bragg peak (SOBP) are still needed for evaluating the risk to normal tissues in close proximity to the irradiated volume. Finally, radiation-induced intercellular bystander signaling has been suggested to play an important role that might significantly affect the outcome of radiation therapy by extending the range over which the radiation damage is induced 25, 26.

Here we have quantified the variations in DNA DSB damage induction and repair along clinically relevant proton beams. AG01522 cells were placed at precise positions along 60-MeV proton beams and the DNA repair kinetics followed up to 24 hours using the well-established phosphorylated p53 binding protein 1(53BP1) foci assay 27, 28. Similarly, we also studied the proton-induced bystander effects in the medium-sharing cells >20 mm away from the irradiated samples. Data have been analyzed as a function of dose, particle fluence, and LET obtained from Monte Carlo simulations. Our findings indicate a significant enhancement in the residual DNA damage only at the distal end of the SOBP. On the other hand, nonsignificant 53BP1 foci induction in the bystander cells suggests reduced risk of late normal tissue toxicity in the non–directly exposed surrounding samples.

## Methods and Materials

### Cell culture

Normal human skin fibroblast (AG01522B) cells were obtained from the Coriell Cell Repository (Camden, NJ) and cultured as previously described (29). Cells grown in the slide flasks were shipped to the experimental beam line in low serum medium containing 20 mM *N*-2-hydroxyethylpiperazine-*N′*-2-ethanesulfonic acid buffer and antibiotics. Upon arrival, the cells were incubated at 37°C until being irradiated (>24 hours) and fixed.

### Irradiation and dosimetry

**Fig. 1:**
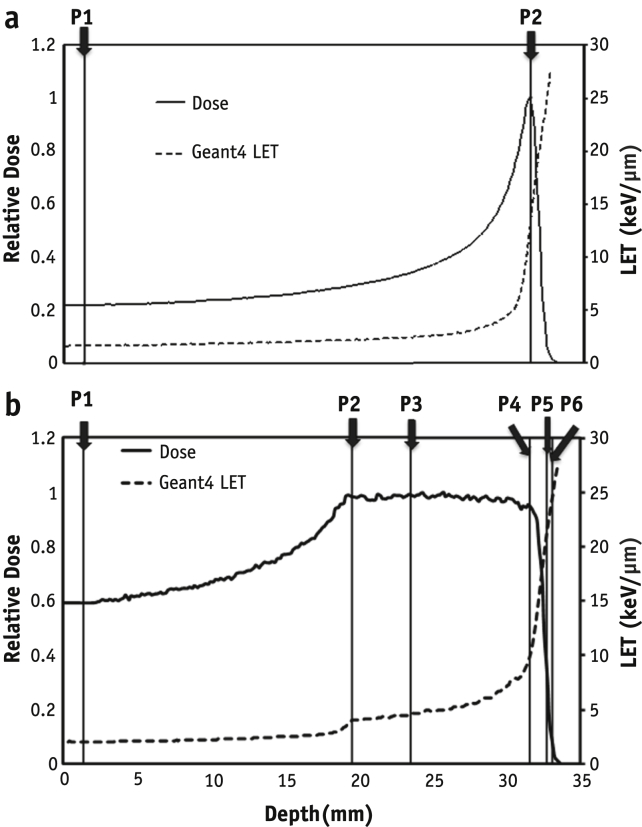
(a) Dose, depth, and linear energy transfer (LET) profiles for monoenergetic and (b) spread-out Bragg peak proton beams. The vertical lines represent the sample irradiation positions. Relative dose across the depth as measured using diode dosimetry is shown using solid lines. Dashed lines indicate linear energy transfer values shown on the secondary *y* axis as calculated using the Geant4 toolkit.

Cells in the slide flasks were exposed to 1 Gy of 60-MeV proton beam generated at the Douglas Cyclotron of the Clatterbridge Cancer Centre and to 225-kVp X rays in our center. Dosimetry was carried out using a Markus chamber as described in reference (30). Details of the irradiation procedure are provided in the Supplementary Information (available online at www.redjournal.org). The depth-dose profiles are shown in Figure 1, where irradiation positions are indicated as P1-P6 in SOBP and P1-P2 for pristine beam. The dose-averaged LET values calculated using the Geant4 toolkit are shown in Supplementary Table 1 (available online at www.redjournal.org).

### 53BP1 foci formation assay

As recommended by seminal reports, the residual 53BP1 assay has been used as a method to quantify clustered DNA damage at the single-cell level 31, 32, 33, 34, 35, 36. After irradiation and incubation for the stipulated time intervals, cells were washed in cold phosphate-buffered saline (PBS) and fixed in 4% paraformaldehyde (Sigma Aldrich, St. Louis, MO) solution in PBS, at room temperature for 20 minutes. Fixed samples were stored in PBS at 4°C. For staining, cells were washed with cold PBS and permeabilized in chilled methanol, washed, and then blocked in 10% goat serum and 0.2% Triton X-100 in PBS, for 1 hour at room temperature. The cells were then probed with 53BP1 primary antibody raised in rabbit (Novus Biologicals, Littleton, CO, USA, Catalog no. NB100-304) at a dilution of 1:1000 for 1 hour at 37°C. Finally the cells were washed, probed at a dilution of 1:1000 with goat anti-rabbit Alexa Flour 488–conjugated secondary antibody (Invitrogen, Life Technologies, Carlsbad, CA, catalog no. A11008 and counterstained with Prolong gold antifade reagent (Invitrogen, Life Technologies, Carlsbad, CA) with 6-diamino-2-phenylindole.

### Image acquisition and data analysis

Images were acquired using a Carl Zeiss (Jena, Germany) Axiovert 200M fluorescence microscope using a ×63 objective. Because of the cell nucleus thickness being comparable to the objective depth of focus, no Z-stack analysis was required. For each data point >100 cells were manually scored randomly in duplicate experiments. The values plotted in graphs are mean ± SEM. Statistical significance was calculated using a 2-tailed unpaired Student *t* test, and *P* values ≤.05 were considered statistically significant.

## Results

### DNA DSB induction and kinetics in response to 60-MeV monoenergetic protons

**Fig. 2:**
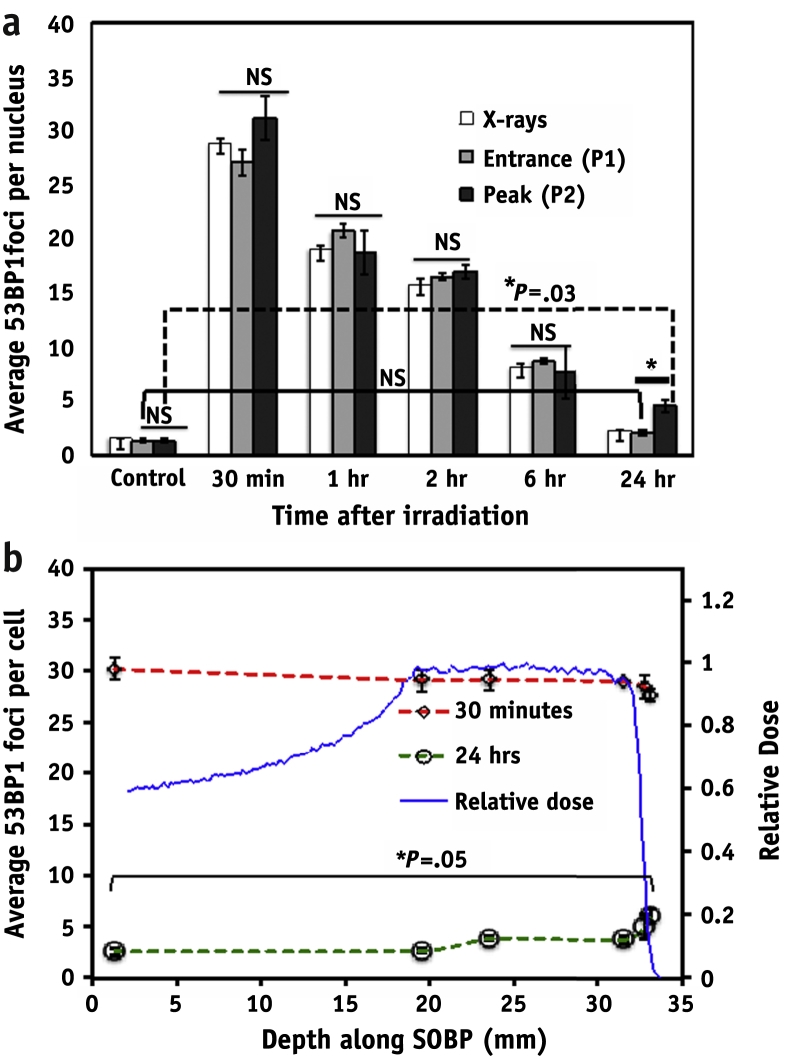
53BP1 foci in AG01522 cells exposed to 1 Gy of (a) 225-kVp X rays, (b) at the beam entrance position (P1) and the Bragg peak (P2) of 60-MeV monoenergetic protons. 53BP1 foci induction and persistence along the spread-out Bragg peak (SOBP) is shown in red at 30 minutes and in green at 24 hours of irradiation. The relative normalized water equivalent dose along the SOBP is shown in blue. Error bars represent ± SEM. Statistical significance was calculated using a 2-tailed, unpaired *t* test, with *P*≤.05 considered as significant. NS = nonsignificant. *Significant. For significance analysis for SOBP data, the average foci numbers of the different positions are compared with the foci number at entrance. A color version of this figure is available at www.redjournal.org.

Figure 2a shows the 53BP1 foci induction in cells directly irradiated with 225-kVp X rays, entrance (P1), and Bragg peak (P2) of the pristine proton beam. The unirradiated controls showed an average number of 1.4 ± 0.19 foci per cell that increased to 28.9 ± 0.37, 27.1 ± 1.25, and 31.1 ± 1.97 in the cells irradiated with 1 Gy of X rays, proton entrance (P1), and Bragg peak (P2) position, respectively, at 30 minutes. No statistically significant differences in the number of foci per cell were observed for all the time points except 24 hours. At 24 hours, although the average foci number per cell at P1 and X rays was similar to that for controls, cells irradiated at the Bragg peak showed significantly (*P*=.03) higher foci numbers (4.6 ± 0.6) than controls (1.4 ± 0.19). This indicates that although most of the DNA lesions are repaired, a small but significant fraction at the Bragg peak position persists >24 hours after irradiation.

### Induction and repair of residual damage along the SOBP

As shown in Figure 2b, nonsignificant variation in foci induction at 30 minutes was observed in cells irradiated along the SOBP. The average number of 53BP1 foci at the entrance (P1) was 30.2 ± 1.10 (1 Gy, fixed at 30 minutes), and similarly the average foci per cell was 29.1 ± 1.7, 29.2 ± 1.1, 28.9 ± 1.1, 28.4 ± 1.2, and 27.6 ± 0.6 foci per cell for the P2-P6 positions, respectively. Twenty-four hours after irradiation, although data indicate an increasing foci trend with depth, only cells irradiated at P6 showed significantly (*P*=.05) increased (6.04 ± 0.65) foci per cell compared with P1.

### Subpopulation radiosensitivity analysis

**Fig. 3:**
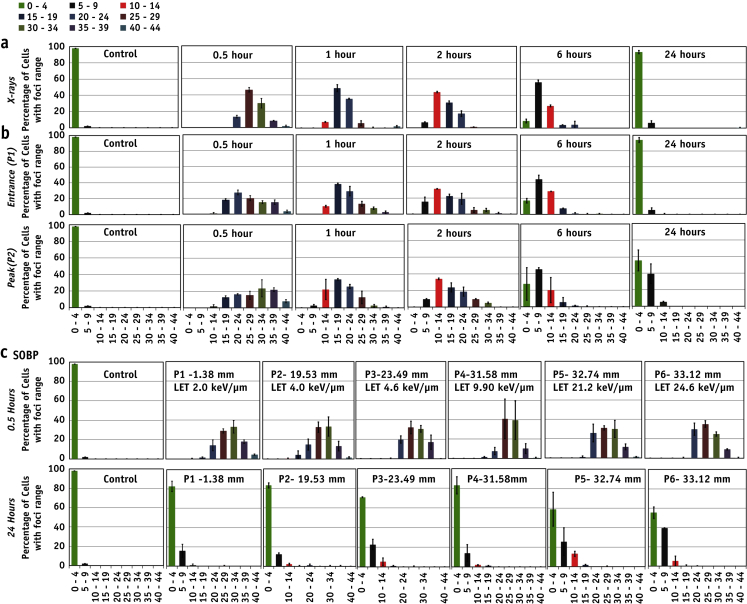
Subpopulation radiosensitivity analysis using distribution of foci per cell for various time points (0.5-24 hours) in the directly exposed cells to (a) 225-kVp X rays, (b) entrance (P1) and the peak (P2) of pristine beam, and (c) along the various depths in the spread-out Bragg peak (SOBP). Range of foci per cell is shown on the *x* axis, and fraction of cells with range of foci is shown on the *y* axis.

Subpopulation radiosensitivity was analyzed using foci per cell distribution patterns. Controls showed >99% of the cells with 0 to 4 foci, whereas 30 minutes after 1 Gy x-ray exposure most of the cells showed approximately 20 to 24 foci (Fig. 3). Foci distribution histograms followed a Gaussian distribution (Supplementary Fig. 2; available online at www.redjournal.org), shifting in time toward lower value of foci per cell. The foci distribution pattern in cells irradiated at the pristine beam entrance position (P1) was similar to the x-ray–induced pattern. However, the foci distribution for the proton-irradiated samples appeared wider than for X rays, possibly indicating inhomogeneous levels of damage at the cellular level despite a very uniform dose delivery at the macroscopic level. This inhomogeneity in levels of DNA damage could be associated with the relatively low number of proton traversals per cell for the delivery of 1 Gy: the estimated particle fluence at the entrance is approximately 520 protons per cell, and at the peak position (P2) is approximately 63 protons per cell (assuming 133 μm^2^ for a typical AG01522 nucleus cross-section and LET value 1.6 and 13.1 keV/μm, respectively, at the entrance and peak).

### DNA DSB in unirradiated medium-sharing bystander cells

**Fig. 4:**
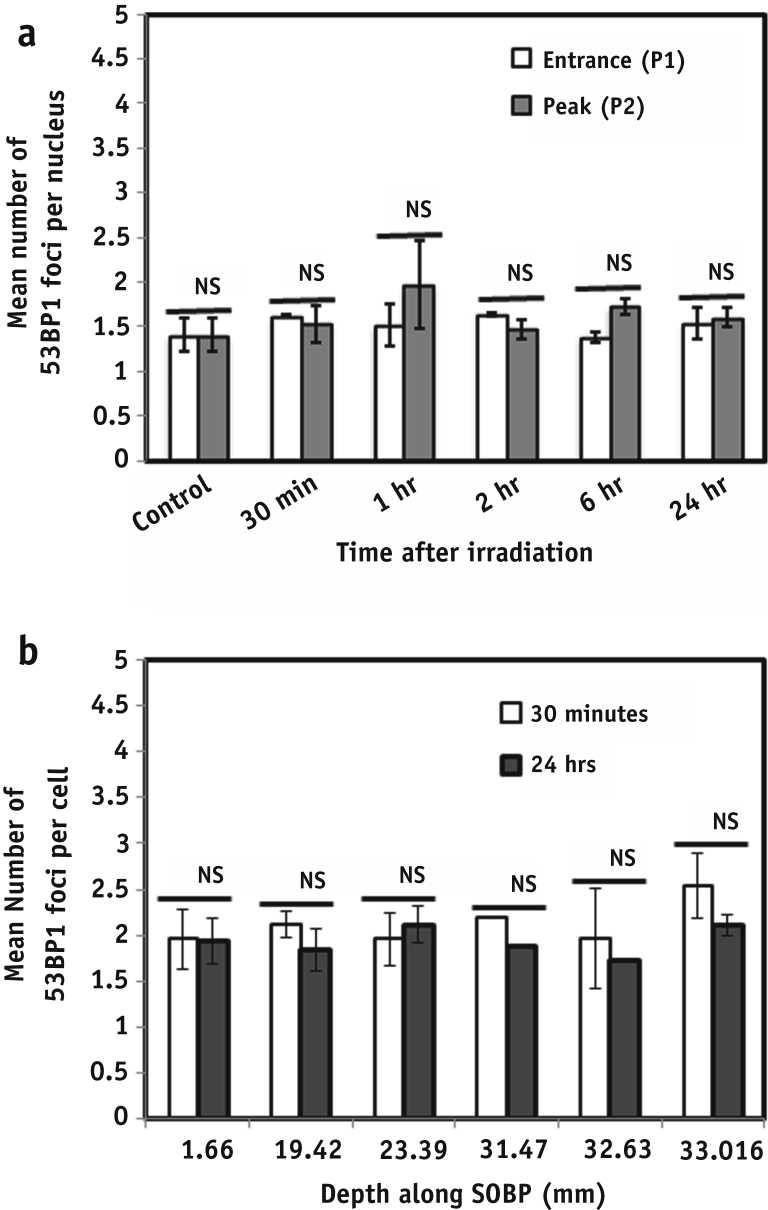
(a) 53BP1 foci induction in the medium-sharing bystander cells at the beam entrance (P1) and peak (P2) position of a 60-MeV monoenergetic beam at various time points. Error bars represent ± SEM of the 2 independent replicates. (b) Residual damage in the medium-sharing cells along the spread-out Bragg peak (SOBP) solid bars indicate the unrepaired foci remaining >24 hours. Statistical significance was calculated using a 2-tailed, unpaired *t* test, with *P*≤.05 considered as significant. NS = nonsignificant. *Significant.

**Fig. 5:**
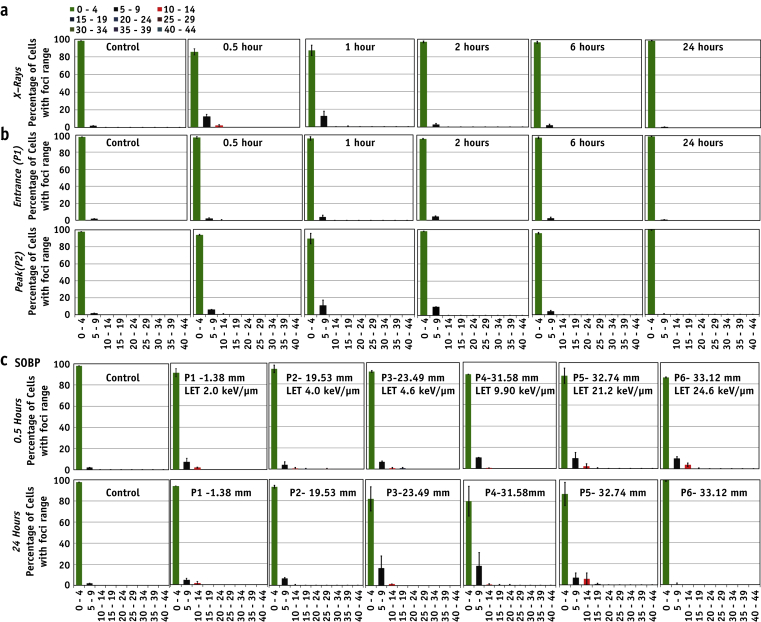
Subpopulation radiosenstivity analysis in medium-sharing bystander cells using foci per cell distribution for various time points (from 0.5-24 hours) after exposure to (a) X rays, (b) entrance (P1) and peak (P2) position of monoenergetic 60-MeV proton beam, and (c) at various depths along the spread-out Bragg peak (SOBP). Range of 53BP1 foci per cell is shown on the *x* axis, and fraction of cells with range of foci is shown on the *y* axis.

DNA DSB repair in unexposed bystander cells approximately 2 cm away from the irradiated cells at the entrance and peak positions was also evaluated. The experiments aimed to investigate the DNA damage caused by bystander factors released into the medium by irradiated cells. Using Geant4 simulations and measurements with Gafchromic films, the scattered dose was found to be negligible at a distance of 2 cm from the irradiation field. Sub-confluent slide flasks were marked into 3 equal parts and one-third irradiated while the rest of the flask was shielded. Figure 4a and b shows the kinetics of 53BP1 foci per cell in the medium-sharing bystander cells. We did not observe any statistically significant differences in the average number of foci in the cells irradiated to 1 Gy of 60 MeV pristine and SOBP beam, irrespective of the position. The foci distribution, however, indicates a small fraction of cells (10%-15%) with bystander foci similar to those observed with directly exposed X rays (Fig. 5).

### Relation between 53BP1 foci induction LET and cell-killing RBE

**Fig. 6:**
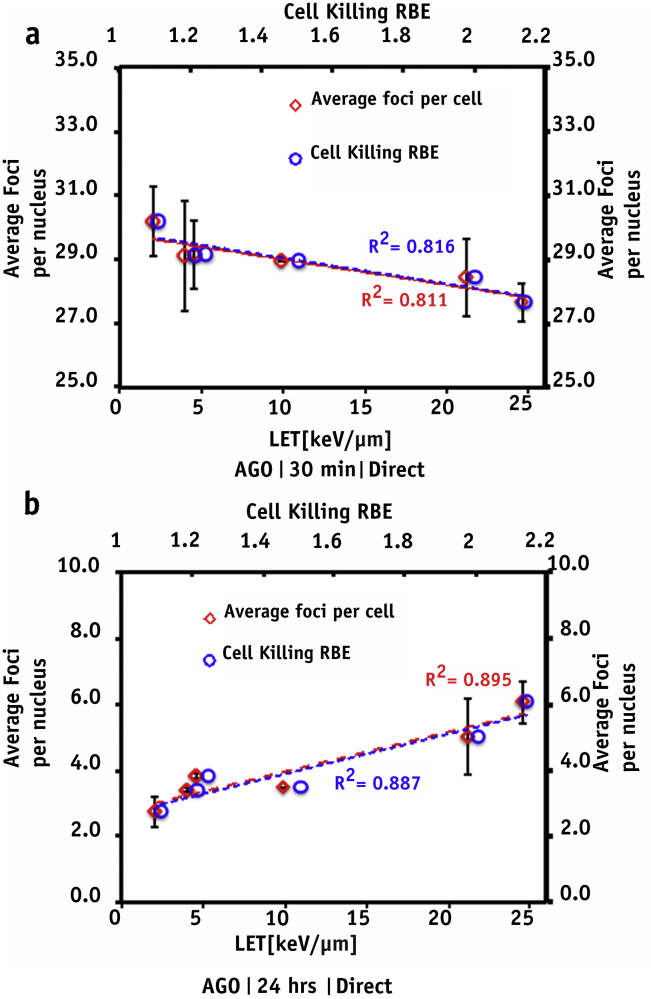
53BP1 foci induction expressed as a function of linear energy transfer (LET) and cell killing relative biological effectiveness (RBE) obtained using the expression: RBE = ((α_x_^2^ + 4 β_x_ D_p_ (α_x_ + λ LET + β_x_ D_p_))ˆ(1/2) – α_x_))/(2 β_x_ D_p_), as published in reference 29. (a) 53BP1 foci induction in cells fixed after 30 minutes (red) along the spread-out Bragg peak and comparison with the predicted RBE (blue circles) and (b) 24 hours as a function of linear energy transfer. Dashed lines indicate the goodness of fit. Error bars represent ±SEM of 2 independent replicates. A color version of this figure is available at www.redjournal.org.

Because DNA DSB damage is well evidenced to play a crucial role in cell killing, we evaluated the relationship between 53BP1 foci, LET, and the RBE for cell killing. Linear energy transfer was obtained using the Geant4 simulation kit, and the RBE was calculated using a parameterized model (29): RBE = ((α_x_^2^ + 4β_x_D_p_(α_x_ + λLET + β_x_D_p_))ˆ(1/2) − α_x_))/(2β_x_D_p_), where α_x_, β_x_ are the α and β parameter from the x-ray exposure, D_p_ is the proton dose, and the λ parameter for AG01522 cells is 0.0451 μm keV^−1^ Gy^−1^. Foci induction kinetics (Fig. 6a, b) across various depths in the SOBP did not show a particularly good correlation with LET (*R*^2^ = 0.81; where *R*^2^ is the coefficient indicating goodness of fit of a function); however, a good correlation (*R*^2^ = 0.91) between the residual foci at 24 hours and LET was obtained. Conversely, medium-sharing bystander cells revealed a good correlation with LET (*R*^2^ = 0.94) at 30 minutes but not at 24 hours (Supplementary Fig. 1; available online at www.redjournal.org). Correlations between foci and RBE are shown in Figure 6a and b for the directly irradiated samples and in Supplementary Figure 1c and d (available online at www.redjournal.org) for the bystander cells.

## Discussion

The debate on variable versus constant RBE in proton therapy treatment planning is still ongoing. Although a constant RBE of 1.1 is currently used with satisfactory results, in vitro and theoretical studies have highlighted the importance and the impact of using variable RBE to fully exploit the potentials of charged particles with strategies such as LET painting of hypoxic areas. Wedenberg and Toma-Dasu (37) recently reported that disregarding RBE variations could lead to less-effective dose plans. Several investigators have also demonstrated a key role of persistent DNA DSBs in late normal tissue radiation toxicity 12, 13, 14 and the selective role of protein kinases (ie, ATM and ATR) in the repair of complex DNA breaks 17, 18, 19, highlighting possible chemical inhibitory strategies for further modulating the biological response along proton beams.

In this study we have shown that even modest variations in LET, as along the proton Bragg curve, can result in different degrees of DNA damage, with cells irradiated at distal positions showing significant persistent 53BP1 foci 24 hours after irradiation. To further elucidate the relationship between LET and induction of residual DNA damage, we studied 53BP1 induction along 6 positions of a clinical 60-MeV SOBP. The average foci per cell number at 30 minutes did not show a significant difference for any of the irradiation positions, indicating a negligible overlapping of foci along the proton track. However, at 24 hours after irradiation, the distal SOBP positions showed significant amounts of residual foci (Fig. 2b). The relative foci induction obtained by dividing the average number of proton-induced foci by the number of x-ray–induced foci at given time points for the pristine and SOBP positions is shown in Supplementary Figure 5 (available online at www.redjournal.org). Further analysis indicated a linear trend (*R*^2^ = 0.9) between residual foci and LET (0-30 keV/μm). Wilkens and Oelfke (38) have reported a linear relationship between RBE and LET up to 100 keV/μm; in our study the highest LET value at the distal end of the SOBP was 24.2 keV/μm, and our findings support this linear trend and highlight the strong link between residual foci and radiobiological response. It is also interesting to note that the number of foci per track per cell follows a linear relationship both at 30 minutes and 24 hours after irradiation (Supplementary Fig. 4; available online at www.redjournal.org). From these data it is possible to extract the minimum LET value required to cause residual DNA damage (LET_crit_ = 2.5 keV/μm). As expected, the foci per cell distribution (Fig. 3) showed a time-dependent shift toward background values. However, although x-ray data follow a normal distribution, the proton distributions seem to be broader and slower for the Bragg peak exposure. This further supports the hypothesis of significant variation in the complexity of DNA breaks induction and repair processes.

The clinical importance of non-targeted or bystander effects has been well reviewed 25, 26, 39. In our study we investigated the contribution of factors released into the medium by directly irradiated cells upon DNA damage in the nonirradiated cells >2 cm away. This distance was chosen to exclude damage induced by secondary scattered particles. Using our current setup we did not detect any significant increase in 53BP1 foci in the medium-sharing bystander cells for either monoenergetic (Fig. 4a) or modulated exposures (Fig. 4b). However, there were differences in the foci per cell distribution (Fig. 5). Whereas the majority (85%-90%) of bystander cells showed 0 to 5 foci, the remaining subgroup showed more than 5 foci per cell, which decreased to 1 to 2 at 24 hours after irradiation. This would suggest that DNA lesions caused in non-directly exposed cells affects only a small subpopulation.

Synthetic lethality-inducing agents such as gemcitabine and cisplatins have been effectively used in cancer treatment owing to their ability to inhibit DNA replication, either by stalling the replication fork or inter-/intrastrand DNA cross-links in the proliferating tumor cells. As reported by Jones et al (40), stalling replication forks also affect DNA repair, causing more persistent DNA DSBs. In vitro (37) and clinical (41) preliminary studies have reported promising results for combined use of cisplatin and poly ADP ribose polymerase inhibitors with proton beams, demanding further investigations regarding the link between quality of damage and the inhibition of repair pathways. Our findings are in agreement with the earlier studies reporting a low- and high-LET behavior for proton beams. The variation of the complexity of DNA breaks along the proton beam seems to be of a significant level for the cellular processes and will have to be taken into account for optimizing proton treatment strategies, particularly in combination with chemotherapeutic agents.
