# Supplementary material for: Variations in the Processing of DNA Double-Strand Breaks Along 60-MeV Therapeutic Proton Beams
Source: Int J Radiat Oncol Biol Phys. 2016 May 1;95(1):86–94. doi: 10.1016/j.ijrobp.2015.07.2279 (PMC4840231; doi:10.1016/j.ijrobp.2015.07.2279)
Supplement: Supplementary Material [file mmc1.pdf]

Supplementary Information

Irradiation and Dosimetry: 1 Gy proton dose (dose rate 4 Gy/min) was delivered to the cells in the slide flasks filled with culture medium and held vertically in the beam path by a custom made X-Y axis translator. Samples were irradiated through the slide flask base using a 60 MeV proton beam generated at the XXX Cyclotron of the YYY Cancer Centre. Water equivalent depths were simulated using PMMA (Polymethyl methacrylate) degraders of varied thicknesses interposed in front of slide flasks. Reference dosimetry was carried out using Markus™ chamber as described by XXX *et al* (32). An 11 mm wide SOBP was generated by introducing a range modulator wheel in the beam-line as routinely used for patients. Further validation of the dosimetry was done by reproducing a qualitative dose-depth profile using Gafchromic (EBT2) film to achieve positional accuracy of ~100 μm with respect to the depth-dose profiles obtained using the Markus chamber, as shown in figure-1, where irradiation positions are indicated as P1-P6 in SOBP and P1 and P2 for pristine beam. The dose averaged LET as a function of depth was calculated using Geant4 Monte Carlo toolkit and values are shown in Table-1. AG01522 in the same passage number and under similar conditions as used in proton irradiation, were irradiated using 225 kVp X- rays (XRAD225, Precision X-Ray Inc.) fitted with a 2 mm thick copper filter at a dose rate of 0.591 Gy/min.

Tables

Position	Water Depth (mm)	LET (keV/µm)
1	1.38	1.7
2	32.50	13.6

Table-1. Characteristic values of depth positions simulated in monoenergetic beam configuration using PMMA beam degraders. LET values were calculated using GEANT4 Monte Carlo simulation tool kit.

Position	Water Depth (mm)	LET (keV/μm)
1	1.38	2.0
2	19.53	4.0
3	23.49	4.6
4	31.58	9.9
5	32.74	21.2
6	33.12	24.6

Table-2. Characteristic values of depth positions simulated in modulated SOBP beam configuration using range modulator wheel. LET values were calculated using GEANT 4 Monte Carlo simulation tool kit.

Figure-1

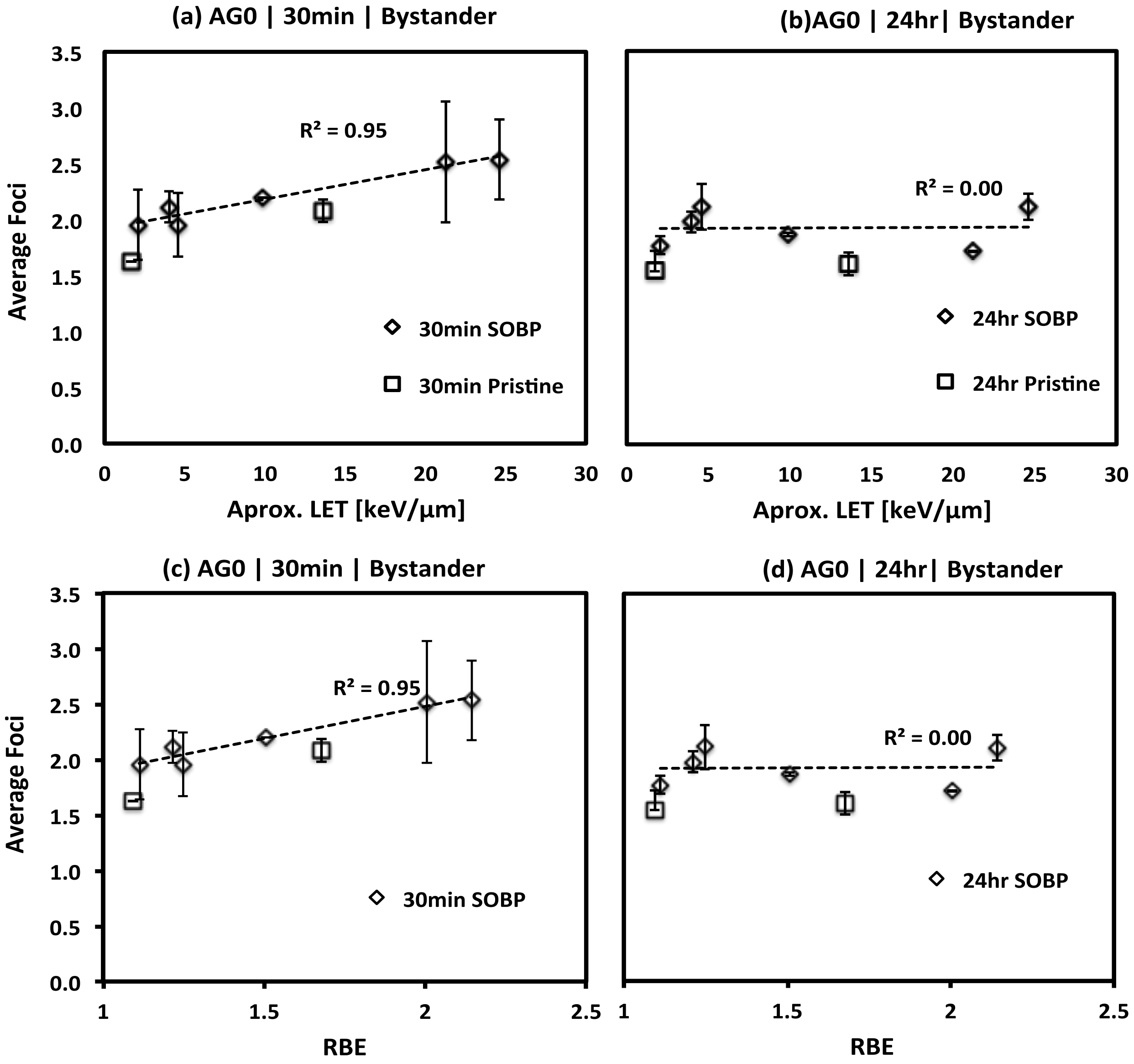

Figure-1. 53BP1 foci and LET relation in the medium sharing bystander cells is shown in (a) at 30 minutes and (b) 24 hours after irradiation. RBE and 53BP1 foci relationship in the cells exposed to 1 Gy of either pristine or SOBP of 60 MeV protons in directly irradiated fixed at 30 minutes (c) and 24 hours after irradiation (d). LET values were obtained through simulations carried out using GEANT 4 Monte Carlo kit for each position at which cells were irradiated. Dashed lines indicate the goodness of fit of the average number of foci per cell as function of

LET. Error bars represent ± standard error of the mean of two independent replicates. Statistical significance was calculated using two-tailed unpaired t-test with $P \leq 0.05$ considered as significant. NS-non-significant, *-significant.

Figure-2

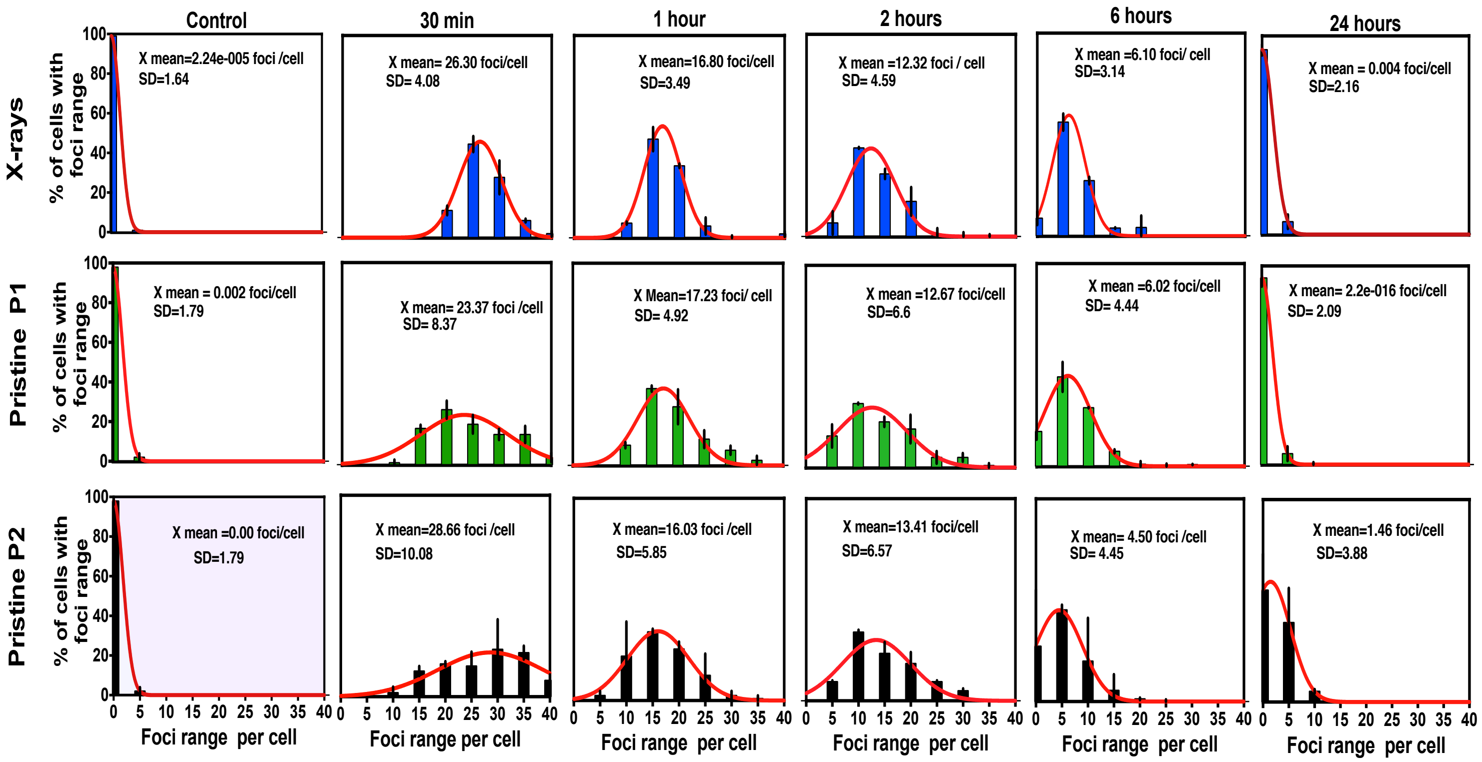

Figure-2. Sub-population radiosenstivity analysis showing the Gaussian distribution of average foci per cell vs the range of foci per cell. Red line indicates the Gaussian fit lines obtained trough the equation: $Y = Amplitude * exp(-0.5 * ((X - Mean) / SD)^2)$

Where best-fit values of the Gaussian distribution are shown by X (mean foci per cell) and SD denotes the standard deviation of mean.

Figure-3.

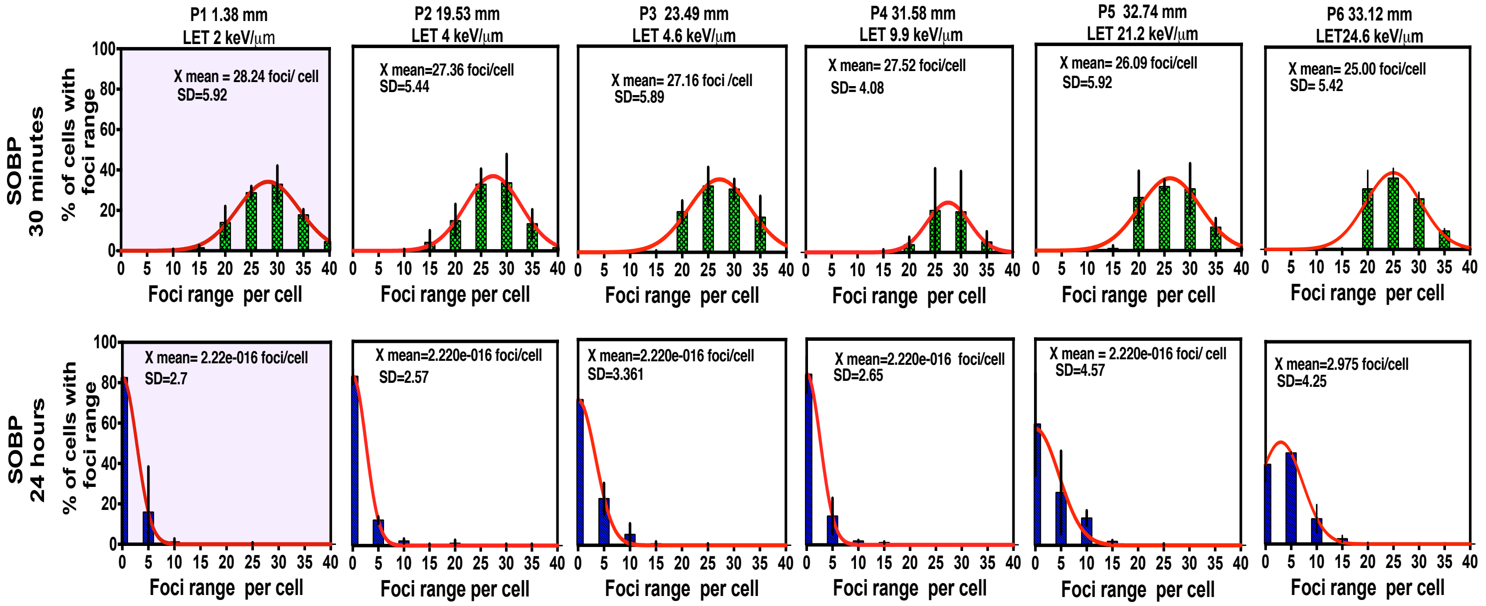

Figure-3. Sub-population radiosenstivity analysis showing the Gaussian distribution of percentage of foci vs average foci per cell induced at 30 minutes and 24 hours after irradiation along the various positions along SOBP. At 30 minutes the average foci per cell followed Gaussian distribution, which could not be noticed at 24 hours post irradiation. Red line indicates the Gaussian fit lines obtained trough the equation:

Equation: Y = Amplitude * exp(-0.5 * ((X - Mean) / SD)^2)

where best fit values of the Gaussian distribution are shown by X (mean foci per cell) and SD denotes the standard deviation of mean.

Figure-4.

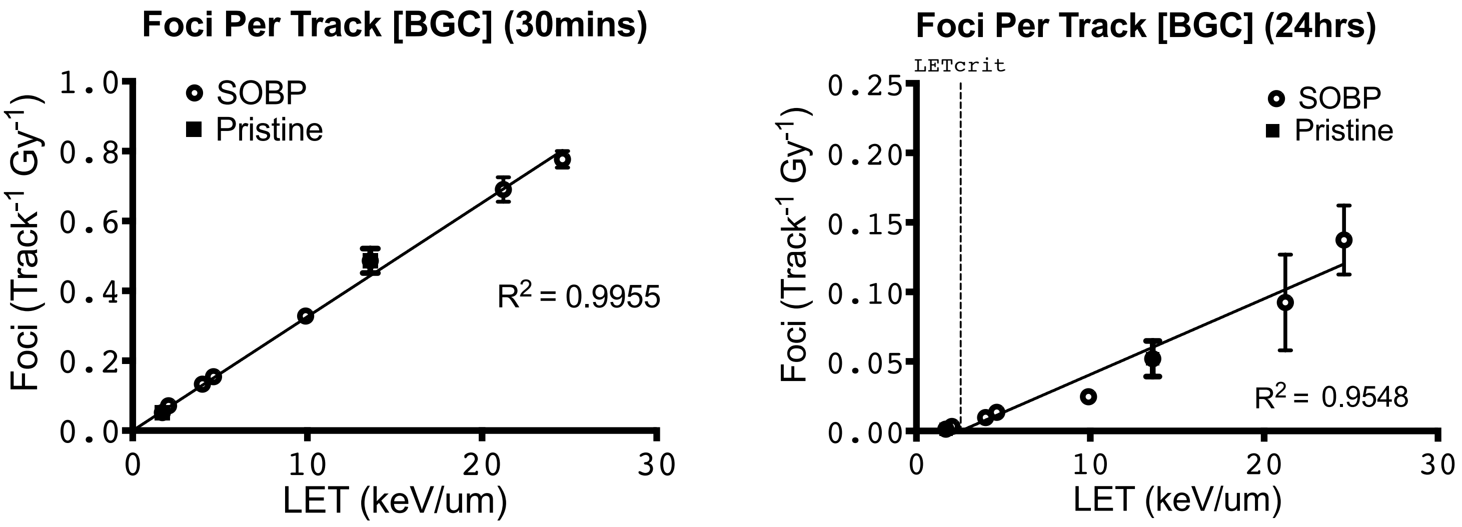

Figure -4. Relationship between number of foci per particle track per cell and particle LET. Extrapolation of the linear fitting to 0 foci provides an estimate of the critical LET for the induction of residual foci. $LET_{crit} = 2.5$ keV/μm

Figure-5

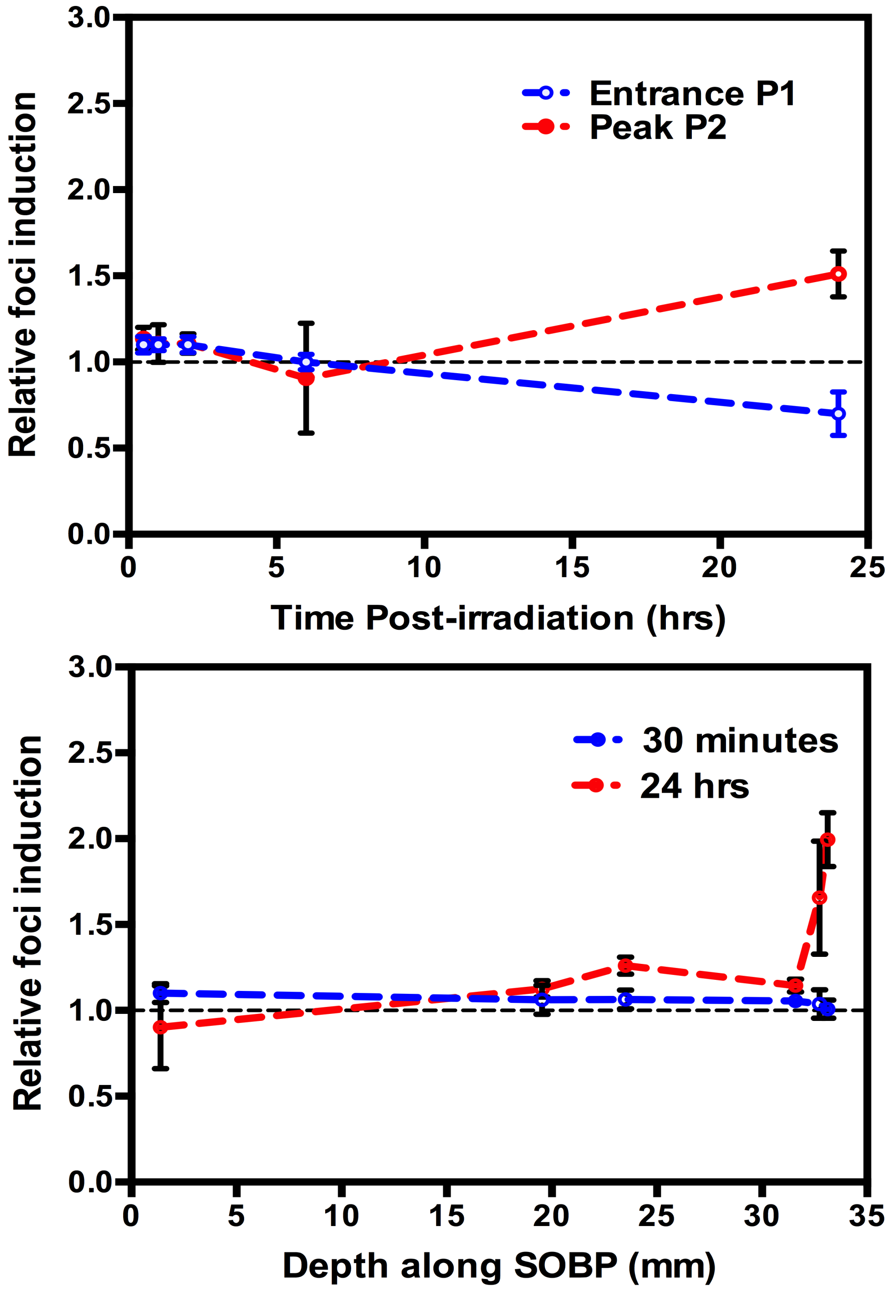

Figure-5. Relative foci induction at various time points after irradiation with 1 Gy of 60 MeV Protons. For Relative foci induction calculation, protons induced average foci number at particular time point is divided by the X-rays induced average foci number at corresponding time points. (A) Relative foci induction at various time points after irradiation where blue indicates foci induction at the entrance, red indicates foci induction at peak and black dashed line is used to indicate value 1 on the Y axis. (B) Relative foci induction along various depths of SOBP in cells fixed at 30 minutes (blue) and 24 hours (red) after irradiation and black dashed line indicates -1 on the Y axis
